# Supplementary material for: Genome-wide polygenic risk impact on intracranial aneurysms and acute ischemic stroke
Source: PLoS One. 2022 Apr 15;17(4):e0265581. doi: 10.1371/journal.pone.0265581 (PMC9012378; doi:10.1371/journal.pone.0265581)
Supplement: S1 Table — (PDF) [file pone.0265581.s001.pdf]

S1 Table. Weighted polygenic risk scores from the genome-wide association study

Gene	Chr	SNP	NR[a]	R[a]	ln(OR)[b]	OR[b]	P[b]
GWA $p < 5 \times 10^{-8}$							
PRDM2	1p36.21	rs61775135	A	C	1.153597	0.3155	3.59×10^{13}
GBA	1q22	rs75822236	C	T	5.083886	161.4	1.09×10^{-19}
FMO4	1q24.3	rs3737926	T	C	1.014731	0.3625	1.83×10^{-10}
RNF144A	2p25.1	rs6741819	T	C	1.382302	0.251	4.05×10^{-14}
FLJ45964	2q37.3	rs59626274	T	C	1.342852	0.2611	5.78×10^{-14}
LINC01237	2q37.3	rs78458145	A	G	1.407722	0.2447	3.14×10^{-15}
SPCS3	4q34.2	rs17688188	A	G	1.312301	0.2692	5.99×10^{-13}
TCF24	8q13.1	rs112859779	T	C	1.686319	0.1852	3.33×10^{-16}
C9orf92	9p22.3	rs12350582	A	G	0.888714	2.432	1.49×10^{-8}
LINGO2	9p21.1	rs56942085	G	A	1.809108	6.105	3.90×10^{-8}
TMOD1	9q22.33	rs1052270	T	C	0.893063	0.4094	2.71×10^{-8}
SUSD1	9q31.3	rs79461840	T	C	1.933693	6.915	1.40×10^{-8}
LINC00474	9q33.1	rs4979583	C	T	0.896496	2.451	4.61×10^{-9}
OLFML2A	9q33.3	rs79134766	A	G	1.972562	0.1391	1.70×10^{-19}
MYEOV	11q13.3	rs76855873	T	C	1.107451	0.3304	1.23×10^{-11}
ARHGAP32	11q24.3	rs371331393	G	A	3.774369	43.57	9.32×10^{-27}
CD163L1	12p13.31	rs138525217	C	T	4.33047	75.98	6.20×10^{-23}
SLC2A14	12p13.31	rs118107419	A	C	1.395537	0.2477	1.44×10^{-14}
DRAM1	12q23.2	rs7964241	A	G	0.910675	2.486	1.95×10^{-8}
CUL4A	13q34	rs74115822	G	A	1.831781	6.245	1.12×10^{-10}
LINC02130	16p13.12	rs11646803	T	C	0.790319	0.4537	4.76×10^{-10}

LOC102724084	16q23.2	rs75861150	C	T	1.934476	0.1445	1.27×10^{-8}
SCARF1	17p13.3	rs3744644	C	G	0.943405	0.3893	6.02×10^{-9}
MINK1	17p13.2	rs72835045	A	G	1.382701	0.2509	1.69×10^{-12}
SLC47A1	17p11.2	rs2440154	G	A	0.981704	2.669	1.42×10^{-8}
NAPA-AS1	19q13.32	rs55800589	C	G	0.963644	0.3815	3.35×10^{-13}
DSCAM	21q22.2	rs727333	A	C	1.364533	0.2555	3.69×10^{-14}
LRRC3	21q22.3	rs116969723	A	G	1.447744	0.2351	3.83×10^{-15}
SLC5A4-AS1	22q12.3	rs117398778	T	C	1.318818	3.739	2.00×10^{-9}
Reported $p < 0.05$							
BOLL	2q33.1	rs700651	A	G	0.34713	1.415	0.00789
EDNRA	4q31.22	rs6841581	A	G	0.630548	0.5323	0.00065

OR, odds ratio; SNP, single-nucleotide polymorphism.

[a]Non-risk (NR) and risk (R) allele were described.

[b]The values that indicates beta coefficient were computed by the natural log transformed OR (i.e., ln(OR)).

[c]OR and *p*-value were estimated by the multivariate logistic regression model after adjusting for age, gender, hypertension, diabetes, hyperlipidemia, smoking status, and four genetic ancestry values.
