# Supplementary material for: Genome-wide polygenic risk impact on intracranial aneurysms and acute ischemic stroke
Source: PLoS One. 2022 Apr 15;17(4):e0265581. doi: 10.1371/journal.pone.0265581 (PMC9012378; doi:10.1371/journal.pone.0265581)
Supplement: S2 Table — (PDF) [file pone.0265581.s002.pdf]

S2 Table. Application of weighted polygenic risk scores according to subtypes of acute ischemic stroke (AIS)

Model[a]	Case, N (%)	Control, N (%)	OR (95% CI)[b]	*P*[b]	Sens.[c]	Spec.[c]	AUROC (95% CI)[c]
CE	N=50	N=296					
T1: 0.290-0.712	9 (18.0)	213 (72.0)	Reference				
T2: 0.712-0.789	27 (54.0)	72 (24.3)	11.13 (3.99-31.03)	4.1×10^{-6}	0.82	0.72	
T3: 0.789-1.126	14 (28.0)	11 (3.7)	83.01 (17.32-397.76)	3.2×10^{-8}	0.28	0.963	0.794 (0.730-0.857)
LAA	N=72						
T1: 0.290-0.712	13 (18.1)		Reference				
T2: 0.712-0.789	38 (52.8)		9.45 (4.31-20.71)	2.0×10^{-8}	0.819	0.72	
T3: 0.789-1.126	21 (29.2)		42.67 (14.25-127.78)	2.0×10^{-11}	0.292	0.963	0.795 (0.741-0.850)
CE	N=50						
T1: 0.290-0.712	9 (18.0)		Reference				
T2: 0.712-0.789	27 (54.0)		11.13 (3.99-31.03)	4.1×10^{-6}	0.82	0.72	
T3: 0.789-1.126	14 (28.0)		83.01 (17.32-397.76)	3.2×10^{-8}	0.28	0.963	0.794 (0.730-0.857)
SVO	N=75						
T1: 0.290-0.712	12 (16.0)		Reference				
T2: 0.712-0.789	41 (54.7)		11.52 (5.03-26.4)	7.6×10^{-9}	0.84	0.72	
T3: 0.789-1.126	22 (29.3)		38.83 (12.30-122.51)	4.3×10^{-10}	0.293	0.963	0.805 (0.754-0.857)
UD	N=25						

T1: 0.290-0.712	2 (8.0)	Reference				
T2: 0.712-0.789	17 (68.0)	35.05 (6.70-183.33)	2.5×10^{-5}	0.838	0.72	
T3: 0.789-1.126	6 (24.0)	63.94 (9.58-426.72)	1.8×10^{-5}	0.284	0.963	0.836 (0.771-0.902)

AUROC, area under the receive operating characteristic curve; CI, confidence interval; OR, odds ratio; Sens., sensitivity; Spec., specificity.

a Weighted polygenic risk model stratified tertile such as lowest risk, middle risk, and highest risk according to subtypes of 222 AIS patients (CE, cardioembolism; LAA, large artery atherosclerosis; SVO, small-vessel occlusion; UD, undetermined) and 296 shared controls.

[b] OR, 95% CI, and *p*-value were estimated by the multivariate logistic regression analyses after adjusting for age, gender, hypertension, diabetes, hyperlipidemia, smoking status, and four principal component values.

[c] Sensitivity, specificity, and AUROC (95% CI) were estimated by "*roctab*" package of STATA software.z
