# Supplementary material for: Genome-wide polygenic risk impact on intracranial aneurysms and acute ischemic stroke
Source: PLoS One. 2022 Apr 15;17(4):e0265581. doi: 10.1371/journal.pone.0265581 (PMC9012378; doi:10.1371/journal.pone.0265581)
Supplement: Attachment — Submitted filename: Revision letter to Reviewer Comments.pdf [file pone.0265581.s003.pdf]

Dear Editor in Chief

Manuscript ID: PONE-D-21-32285

"Genome-wide polygenic risk impact on the intracranial aneurysm and acute ischemic stroke"

We are submitting a revised version of the above manuscript according to the letter from the Editorial Committee. We have made corrections and clarifications in the manuscript based on reviewers' comments. In this revision manuscript, we inserted two additional authorships (JJL and DN) because those coauthors contributed to data curation of stroke subtypes and writing to the review process. We highlighted the revised text in gray color in the revised manuscript (filename: Revised Manuscript.docx), the revised manuscript without gray highlighted (filename: Manuscript.docx), and attached the supplementary material (filename: Online_Supplemental_Data.pdf) and the updated figures corrected by PACE (https://pacev2.apexcovantage.com/) (i.e., filenames: Fig1 to Fig4.tif).

Comments from the reviewer 1

Comment 1: In this manuscript the authors aim to shed light on hypothetical shared genetic risk factors for Intracranial Aneurysm (IA) and Acute Ischemic Stroke (AIS), presenting and analysing risk models based on weighted Polygenic Risk Score (wPRS) derived from Genome-Wide Association Studies (GWAS) previously published by the same authors. For what is in my competence, the experimental design sounds carefully conceived and the manuscript is well written. Statistics are described in details and conclusions are reported in a clear and appropriate fashion. However, as the same authors stated in the Discussion section, I must observe that the study has limitations, i.e. small sample size and lack of an investigation on associations between AIS subtypes and IA, which could be of major interest in order to implement the predictive power of the analysis. This study, on his current form, provides a

promising starting point and it can have an impact in terms of designing broader and deeper investigations.

On the other hand, I think that the discussion addresses relevant topics, such as the importance of the introduction of PRS derived models to improve risk stratification and the lack of generalizability to non-European ancestry population of existing models. A larger investment on the collection of studies from non-European ancestry is definitely needed.

Answer: Thank you very much for the positive comments on our study. However, as you mentioned, the main limitations are that the sample size in this study was underpowered and the wPRS assessments according to the ischemic stroke subtype based on a large number of patients is an ongoing project. Due to the nature of bioinformatics, if the sample size of the main data in a GWAS is insufficient, the study outcome will be underpowered. Realistically, a way to address this issue is to reduce the false positives associated with diseases by adding a fine-mapping analysis. However, fine-mapping analysis is an alternative approach to discover candidate variants associated with complex traits based on GWAS summary statistics (Reference 1 below). Thus, the best solution would be to increase the number of patients with IA in a future study. Regarding the second limitation, we performed a subsequent analysis of wPRS assessments in the subtypes of acute ischemic stroke (AIS) including cardioembolism (CE, n= 50), large artery atherosclerosis (LAA, n = 72), small-vessel occlusion (SVO, n = 75), and undetermined (UD, n = 25) (Supplemental Table S2 below). Overall, IA-predicting wPRSs increased the risk of all subtypes of AIS with predictabilities between 0.794 and 0.836. Nevertheless, the sample size in each type of stroke was also small. Thus, additional analysis of a large number of AIS patients is necessary. We included these limitations in the Discussion section (page 10 and lines 203-214).

Reference

1. Schaid DJ, Chen W, Larson NB. From genome-wide associations to candidate causal variants by statistical fine-mapping. Nat Rev Genet. 2018;19(8):491-504. http://doi.org/10.1038/s41576-018-0016-z PMID: 2984461

S2 Table. Application of weighted polygenic risk scores according to subtypes of acute ischemic stroke (AIS)

Model[a]	Case, N (%)	Control, N (%)	OR (95% CI)[b]	*P*[b]	Sens.[c]	Spec.[c]	AUROC (95% CI)[c]
CE	N=50	N=296					
T1: 0.290-0.712	9 (18.0)	213 (72.0)	Reference				
T2: 0.712-0.789	27 (54.0)	72 (24.3)	11.13 (3.99-31.03)	4.1×10^{-6}	0.82	0.72	
T3: 0.789-1.126	14 (28.0)	11 (3.7)	83.01 (17.32-397.76)	3.2×10^{-8}	0.28	0.963	0.794 (0.730-0.857)
LAA	N=72						
T1: 0.290-0.712	13 (18.1)		Reference				
T2: 0.712-0.789	38 (52.8)		9.45 (4.31-20.71)	2.0×10^{-8}	0.819	0.72	
T3: 0.789-1.126	21 (29.2)		42.67 (14.25-127.78)	2.0×10^{-11}	0.292	0.963	0.795 (0.741-0.850)
SVO	N=75						
T1: 0.290-0.712	12 (16.0)		Reference				
T2: 0.712-0.789	41 (54.7)		11.52 (5.03-26.4)	7.6×10^{-9}	0.84	0.72	
T3: 0.789-1.126	22 (29.3)		38.83 (12.30-122.51)	4.3×10^{-10}	0.293	0.963	0.805 (0.754-0.857)
UD	N=25						
T1: 0.290-0.712	2 (8.0)		Reference				
T2: 0.712-0.789	17 (68.0)		35.05 (6.70-183.33)	2.5×10^{-5}	0.838	0.72	
T3: 0.789-1.126	6 (24.0)		63.94 (9.58-426.72)	1.8×10^{-5}	0.284	0.963	0.836 (0.771-0.902)

AUROC, area under the receiver operating characteristic curve; CI, confidence interval; OR, odds ratio; Sens., sensitivity; Spec., specificity.

[a] Weighted polygenic risk model stratified by tertiles of lowest risk, middle risk, and highest risk according to the subtypes of 222 AIS patients (CE, cardioembolism; LAA, large artery atherosclerosis; SVO, small-vessel occlusion; UD, undetermined) and 296 shared controls.

[b] OR, 95% CI, and *p*-value were estimated by multivariate logistic regression analysis after adjusting for age, gender, hypertension, diabetes, hyperlipidemia, smoking status, and 4 principal component values.

[c] Sensitivity, specificity, and AUROC (95% CI) were estimated using the *roctab* package in STATA software.

Comments from the reviewer 2

Comment 1: The Authors claim to determine whether the polygenic risk score developed from intracranial aneurysm patients has a common genetic basis with acute ischemic stroke in a Korean population. To do that they applied a weighted PRS model based on a previous genome wide GWAS study using 250 intracranial aneurysm patients in hospital-based multicenter cohort and a validation study in 222 patients who suffered acute ischemic stroke. The work of the Authors is interesting and points out a different way to approach these conditions, which are known to share several clinical risk factors. However, to the date, due to the small size of the sample and the lack of identification of acute ischemic stroke subtypes the use of PRS in common clinical practice isn't feasible yet. Some typos should be revised. Nevertheless, Authors' work is worthy of further investigation on a larger cohort which a more accurate clinical subtyping.

Answer: Per your comments, the relatively small sample size in the study and the absence of an analysis of the acute ischemic stroke (AIS) subtypes by PRS were acknowledged as the main limitations of the study. Due to the nature of bioinformatics, if the sample size of the main data in a GWAS is insufficient, the study outcomes will be underpowered. Realistically, a way to address this issue is to reduce the false positives associated with diseases by adding a fine-mapping analysis. Nevertheless, fine-mapping analysis is an alternative approach to discover candidate variants associated with complex traits based on GWAS summary statistics (Reference 1 below). Thus, the best solution would be to increase the number of patients with IA in a future study. Regarding the second limitation, we performed a subsequent analysis further of wPRS assessments in the subtypes of acute ischemic stroke (AIS) including cardioembolism (CE, n= 50), large artery atherosclerosis (LAA, n = 72), small-vessel occlusion (SVO, n = 75), and undetermined (UD, n = 25) (Supplemental Table S2, below). Overall, IA-predicting wPRSs increased the risk of all subtypes of AIS with predictabilities between 0.794

and 0.836. Nevertheless, the sample size in each stroke subtypes was also small. Thus, additional analysis of a larger number of AIS patients is necessary. We included these limitations in the Discussion section (page 10 and lines 203-214).

Furthermore, English grammar and typographical errors were checked again by English proofreading performed by the native speakers and scientific expertise.

.

Reference

1. Schaid DJ, Chen W, Larson NB. From genome-wide associations to candidate causal variants by statistical fine-mapping. Nat Rev Genet. 2018;19(8):491-504. http://doi.org/10.1038/s41576-018-0016-z PMID: 2984461

S2 Table. Application of weighted polygenic risk scores according to subtypes of acute ischemic stroke (AIS).

Model[a]	Case, N (%)	Control, N (%)	OR (95% CI)[b]	P^b	Sens.[c]	Spec.[c]	AUROC (95% CI)[c]
CE	N=50	N=296					
T1: 0.290-0.712	9 (18.0)	213 (72.0)	Reference				
T2: 0.712-0.789	27 (54.0)	72 (24.3)	11.13 (3.99-31.03)	4.1×10^{-6}	0.82	0.72	
T3: 0.789-1.126	14 (28.0)	11 (3.7)	83.01 (17.32-397.76)	3.2×10^{-8}	0.28	0.963	0.794 (0.730-0.857)
LAA	N=72						
T1: 0.290-0.712	13 (18.1)		Reference				
T2: 0.712-0.789	38 (52.8)		9.45 (4.31-20.71)	2.0×10^{-8}	0.819	0.72	
T3: 0.789-1.126	21 (29.2)		42.67 (14.25-127.78)	2.0×10^{-11}	0.292	0.963	0.795 (0.741-0.850)
SVO	N=75						
T1: 0.290-0.712	12 (16.0)		Reference				
T2: 0.712-0.789	41 (54.7)		11.52 (5.03-26.4)	7.6×10^{-9}	0.84	0.72	
T3: 0.789-1.126	22 (29.3)		38.83 (12.30-122.51)	4.3×10^{-10}	0.293	0.963	0.805 (0.754-0.857)
UD	N=25						
T1: 0.290-0.712	2 (8.0)		Reference				
T2: 0.712-0.789	17 (68.0)		35.05 (6.70-183.33)	2.5×10^{-5}	0.838	0.72	
T3: 0.789-1.126	6 (24.0)		63.94 (9.58-426.72)	1.8×10^{-5}	0.284	0.963	0.836 (0.771-0.902)

AUROC, area under the receive operating characteristic curve; CI, confidence interval; OR, odds ratio; Sens., sensitivity; Spec., specificity.
[a] Weighted polygenic risk model stratified tertile such as lowest risk, middle risk, and highest risk according to subtypes of 222 AIS patients (CE, cardioembolism; LAA, large artery atherosclerosis; SVO, small-vessel occlusion; UD, undetermined) and 296 shared controls.
[b] OR, 95% CI, and *p*-value were estimated by the multivariate logistic regression analyses after adjusting for age, gender, hypertension, diabetes, hyperlipidemia, smoking status, and four principal component values.
[c] Sensitivity, specificity, and AUROC (95% CI) were estimated by "*roctab*" package of STATA software.
